# The Daily Consumption of Cola Can Determine Hypocalcemia: A Case Report of Postsurgical Hypoparathyroidism-Related Hypocalcemia Refractory to Supplemental Therapy with High Doses of Oral Calcium

**DOI:** 10.3389/fendo.2017.00007

**Published:** 2017-01-26

**Authors:** Valentina Guarnotta, Serena Riela, Marina Massaro, Sebastiano Bonventre, Angela Inviati, Alessandro Ciresi, Giuseppe Pizzolanti, Salvatore Benvenga, Carla Giordano

**Affiliations:** ^1^Biomedical Department of Internal and Specialist Medicine (DIBIMIS), Section of Endocrine-Metabolic Diseases, University of Palermo, Palermo, Italy; ^2^Department of Science and Technologies Biological Chemical and Pharmaceutical (STEBICEF), Section of Chemistry, University of Palermo, Palermo, Italy; ^3^Department of General, Emergency and Transplant Surgery, Unit of General and Emergency Surgery, P. Giaccone Policlinico, Palermo, Italy; ^4^Department of Clinical and Experimental Medicine, Endocrinology, University of Messina, Messina, Italy

**Keywords:** hypocalcemia, cola, hypoparathyroidism, hyperphosphatemia, calcium absorption

## Abstract

The consumption of soft drinks is a crucial factor in determining persistent hypocalcemia. The aim of the study is to evaluate the biochemical mechanisms inducing hypocalcemia in a female patient with usual high consumption of cola drink and persistent hypocalcemia, who failed to respond to high doses of calcium and calcitriol supplementation. At baseline and after pentagastrin injection, gastric secretion (Gs) and duodenal secretion (Ds) samples were collected and calcium and total phosphorus (P_tot_) concentrations were evaluated. At the same time, blood calcium, P_tot_, sodium, potassium, chloride, magnesium concentrations, and vitamin D were sampled. After intake of cola (1 L) over 180 min, Gs and Ds and blood were collected and characterized in order to analyze the amount of calcium and P_tot_ or sodium, potassium, magnesium, and chloride ions, respectively. A strong pH decrease was observed after cola intake with an increase in phosphorus concentration. Consequently, a decrease in calcium concentration in Gs and Ds was observed. A decrease in calcium concentration was also observed in blood. In conclusion, we confirm that in patients with postsurgical hypoparathyroidism, the intake of large amounts of cola containing high amounts of phosphoric acid reduces calcium absorption efficiency despite the high doses of calcium therapy.

## Introduction

The consumption of soft drinks, full of phosphoric acid, is a potential factor determining hypocalcemia, notably in patients with hypoparathyroidism. Postoperative hypoparathyroidism and subsequent hypocalcemia are the most frequent complications of total thyroidectomy. Generally, postoperative hypoparathyroidism is associated with transient hypocalcemia. Indeed about 60–70% of cases of postoperative hypocalcemia resolve within 4–6 weeks after surgery, while about 2–10% of patients develop chronic hypoparathyroidism and hypocalcemia (1). Only a small proportion of thyroidectomized patients receiving supplemental calcium therapy remains hypocalcemic (2, 3). Some patients’ treatment fails to respond to calcium supplementation because of unrecognized celiac disease or, less frequently, through unknown causes.

In both the clinical (4, 5) and experimental settings (6), heavy consumption of cola soft drinks is associated with hypocalcemia, and with increased risk of bone fractures (7–9). Indeed, it is well known that in patients with normally functioning parathyroids, oral or parenteral phosphate intake can decrease serum calcium levels by reducing calcium intestinal absorption or increasing calcium excretion (10).

Therefore, patients with postoperative hypoparathyroidism may appear to be more fragile than other patients, if exposed to high doses of cola.

Here, we describe the case of a patient with postsurgical hypoparathyroidism-related hypocalcemia who was treated with high doses of oral calcium and calcitriol supplements, without reaching a good control of calcium levels.

## Background

A 28-year-old woman was hospitalized in our Section of Endocrinology with severe and recurrent hypocalcemic crises (12 times/year). She had undergone total thyroidectomy 8 months before our observation, with postsurgical hypoparathyroidism. She was treated with oral calcium carbonate (10 g/day), calcitriol (2 μg/day), and levo-thyroxine (125 μg/day). Serum total calcemia and phosphoremia were 6 and 5 mg/dL, respectively. Renal, hepatic functions, and serum electrophoretogram were normal. Urinary calcium and phosphorus ions were increased, while calcitonin levels were detectable, due to the persistence of a minimal micro thyroid tissue of 5 mm (Table 1). Celiac disease was excluded based on negativity for serum antibodies (anti-tissue transglutaminase and anti-endomysial), small bowel biopsy, and genetic testing (human leukocyte antigen test). Attempts to normalize calcemia with other calcium formulations (calcium carbonate plus gluconate, calcium lactate, and calcium citrate) were unsuccessful.

**Table 1:** **Baseline clinical and biochemical parameters at first observation and after the interruption of cola’s intake**.

Parameters	Baseline	After cola’s interruption
Weight (kg)	66	67
BMI (kg/m^2^)	25.2	25.6
Urea (mg/dL)	24	34
Creatinine (mg/dL)	0.9	0.8
Na^+^ (mEq/L)	138	140
K^+^ (mEq/L)	3.9	4.7
Cl^+^ (mEq/L)	98	105
Ca^2+^ (mg/dL)	7	9,4
P (mg/dL)	6.5	4.7
Mg^2+^ (mg/dL)	1.6	1.7
Albumin (g/dL)	4.1	4.2
Total proteins (g/dL)	7.2	7.4
25 hydroxy vitamin D (ng/mL)	9.7	15.6
Parathyroid hormone (pg/mL)	1	1
Alkaline phosphatase (U/L)	16	19.6
Osteocalcin (ng/mL)	15	12
Glycemia (mg/dL)	84	78
Urinary calcium/24 h (g/day)	45	30
Urinary phosphorus/24 h (g/day)	70	29
AST (U/L)	15	14
ALT (U/L)	16	18
Beta C-terminal telopeptide (ng/mL)	0.2	0.2
TSH (μU/mL)	2.1	1.8
FT4 (ng/dL)	0.9	1
FT3 (pg/mL)	3.5	3.9
Calcitonin (pg/mL)	1.9	1.6

Careful evaluation of the patient’s history revealed satisfactory compliance with the medical treatment but also habitual heavy daily cola consumption (about 2 L/day). We attempted a complete withdrawal of cola for 2 weeks, and the patient restored normal concentrations of both calcium and phosphorus ions in serum and urine (Table 1). Accordingly, the daily dose of calcium carbonate and calcitriol was decreased from 10 to 5 g/day and from 2 to 1 μg/day, respectively.

To understand the reasons for this significant decrease in calcium supplementation treatment, we designed a protocol evaluating the effect of oral diet cola intake on calcium absorption in the stomach and duodenum. This study was carried out in accordance with the recommendations of the University of Palermo/Policlinico Paolo Giaccone committee with written informed consent from the subject. Patient gave written informed consent in accordance with the Declaration of Helsinki and the recommendations of the University of Palermo/Policlinico Paolo Giaccone committee.

After an overnight fast, multilumen manometric probes in combination with an open-end tip were endoscopically placed *via* the nasal passage to take gastric secretion (Gs) and duodenal secretion (Ds). The baseline Gs pH was 2.6.

An hour before the experimental procedure, a single dose of calcium carbonate (2 g) was administered orally to the patient. At baseline, 10 mL of Gs and Ds samples were obtained, and calcium and total phosphorus (P_tot_ = H_3_PO_4_, ${\text{H}}_{2}{\text{PO}}_{4}^{-}$, ${\text{HPO}}_{4}^{2-}$, and ${\text{PO}}_{4}^{3-}$) concentrations were evaluated. In addition, the calcium, P_tot_, sodium, potassium, magnesium, chloride, and vitamin D concentrations in the blood were evaluated.

Pentagastrin (6 µg/kg) was injected to stimulate Gs. After 5 and 30 min, Gs and blood samples were collected and the concentrations of calcium, P_tot_, sodium, potassium, magnesium, and chloride ions were estimated.

**Figure 1:**
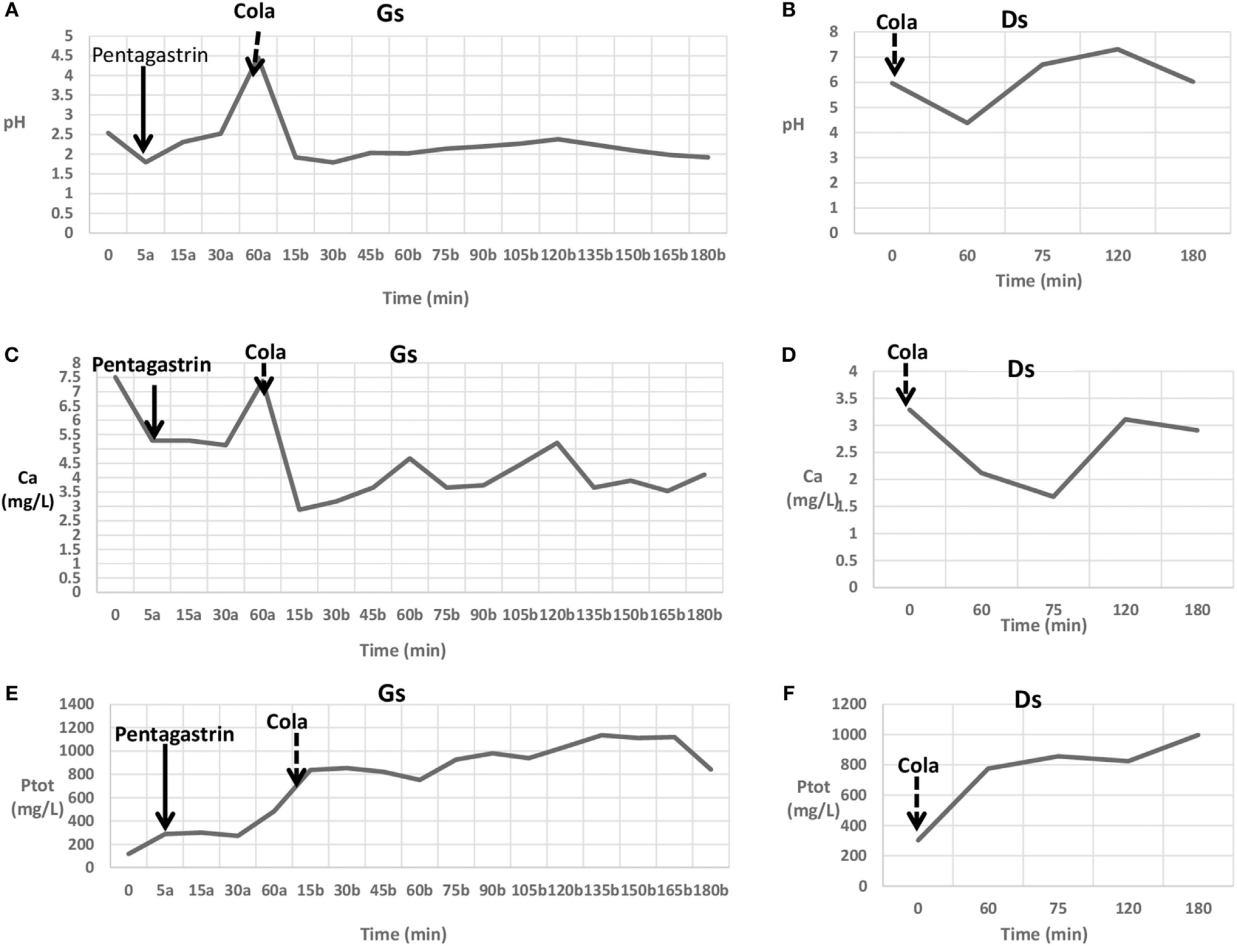
**Gastric secretion (Gs) analysis during pentagastrin stimulation (a) and cola intake (b)**. **(A)** pH changes in gastric content. **(C)** Ca changes in gastric content. **(E)** Total phosphorus (P_tot_) changes in gastric content. Duodenal secretion (Ds) changes before (time 0) and after cola intake. **(B)** pH changes in duodenal content. **(D)** Ca changes in duodenal content. **(F)** P_tot_ changes in duodenal content.

Afterward, the patient orally took 1 L of diet cola, without sugar, to avoid the simulation of endogenous insulin secretion and intracellular phosphate shift, over 180 min (330 mL every 60 min). Gs was drawn from the stomach every 15 min over 180 min after administration of cola, whereas Ds was collected at times 60, 75, 120, and 180 min. In addition, blood samples were obtained every 30 min over 180 min (Figure 1). At the end of the procedure, a urine sample was collected for determination of the urinary calcium and phosphorus.

The day after, pH monitoring was done by placing a pH probe (5 cm) above the upper border of the manometrically determined lower esophageal sphincter.

In basal conditions, serum calcium, phosphorus, magnesium, sodium, potassium, chloride, glycemia, and urinary calcium and phosphorus were in the normal range (results not shown), as were the gastric and duodenal P_tot_ levels (Figure 1). A pentagastrin injection caused a slight decrease in pH after 5 min followed by an increase over 60 min (Figure 1). No differences were observed for serum and urinary parameters (results not shown). A slight change in gastric calcium and phosphorus was observed from 5 to 30 min after a pentagastrin injection, whereas a remarkable increase in P_tot_ level was observed after 60 min (Figure 1).

Cola drinking caused a strong decrease in pH value (Figure 1), and both in stomach and in duodenum, a strong reduction of calcium concentration and a concomitant increase of phosphorus concentration (Figure 1) were detected.

A similar trend was observed in serum, namely a decrease in calcium concentration (9.4 vs. 8.0 mg/dL) and an increase in the phosphorus one (3.8 vs. 5.6 mg/dL). In addition, increases in the calcium/creatinine ratio (29.2 vs. 48.4) and phosphorus/creatinine ratio (67.8 vs. 109.8) were observed. No differences in sodium, potassium, chloride, magnesium, and glycemia levels were detected (results not shown).

Slight but not significant gastroduodenal reflux was detected by the pH meter.

## Discussion

This report describes a case of a patient with severe hypocalcemia secondary to iatrogenic hypoparathyroidism, who because of cola consumption was not responsive to high doses of oral calcium and calcitriol supplementation.

As is well known, calcium is generally absorbed by two general mechanisms in the small intestine. The transcellular active process, located in the duodenum and upper jejunum, involves three major steps: calcium entry across the brush border by calcium transport protein (CaT1); intracellular diffusion, mediated largely by calbindin; and extrusion, mediated by calcium pumps ATPase dependent calcium pumps. The paracellular, passive mechanism occurs in the entire intestine. When calcium intake is low, transcellular calcium transport accounts for a large fraction of the absorbed calcium. When calcium intake is high, transcellular transport accounts for a small part of the absorbed calcium, because CaT1 and calbindin are downregulated (11).

It has been reported that the precipitation of calcium phosphate salts is initiated by a reaction of the calcium and hydrogen phosphate ions, leading to calcium hydrogen phosphate (12–14). The maximal product of the molar concentrations of these ions, which can exist in solution without precipitation, defines their solubility product. When this solubility product exceeds the normal value, precipitation occurs. The solubility product for calcium hydrogen phosphate has been estimated *in vitro* under physiological conditions of temperature, ionic strength, pH, and comparable Ca/P molar concentration ratios (11, 12), and the estimates range from 2.4 to 2.5 × 10^−6^ mol/L.

As is well known, cola represents a strong exogenous source of phosphate, due to high phosphorus content (about 15–20 mg/dL), more than other carbonated soft drinks (15). In this case, the consumption of cola (330 mL every 60 min) caused a sudden change in the pH gastric values as a consequence of the strong cola acidity (pH 1.8). The effect of cola on the calcium concentration seems to be connected to its high phosphate concentration. Indeed, when the cola drink was ingested a considerable decrease in the calcium concentration and, of course, an increase in the phosphorus concentration was observed. After this effect, due to the formation of calcium phosphate species, an increase in both calcium and phosphorus concentrations was detected, probably due to slow release (dissociation) of calcium and phosphorus species. The intake of a second (after 60 min) and third (after 120 min) cola drink caused a qualitatively comparable effect on the calcium and phosphorus ions. Notably, only a small concentration of Ca^2+^ ions is available during the consumption of cola. These effects were observed both in stomach and in duodenum. Indeed, the intestinal content variations largely reflect those of the stomach.

Unexpectedly, a small pH variation was observed during the experimental procedure. Indeed, after the first significant change from 2.54 up to 1.92, the pH value seems to fluctuate around 2.2 ± 0.2.

A comparison between the gastric calcium and phosphorus levels after pentagastrin stimulation and cola intake might suggest that cola has the effect of slowing down the reset of the initial calcium concentration and increasing phosphorus levels, more than pentagastrin (Figure 1). Consequently, the low intestinal calcium absorption caused a sudden decrease in serum calcium levels and an increase in phosphorus serum levels. As expected, the absence of parathyroid glands caused a sharp increase in tubular phosphate reabsorption during cola intake.

Our data show that phosphorus ions lower serum calcium levels by a simple physicochemical precipitation of calcium hydrogen phosphate as its solubility product exceeds the normal value.

Other previous reports described a significant effect of cola on bone (9). Indeed, diets full of phosphorus and low in calcium lead to complexes that reduce serum calcium, stimulating PTH, which, in turn, causes bone resorption. High dietary phosphorus has been demonstrated to cause bone loss in animals (16). In addition, in another study, cola was given to ovariectomized rats with subsequent hypocalcemia and loss of bone mineral density. However, a limit of the current study is not to have evaluated the direct effect of caffeine on hypocalcemia, using a decaffeinated cola. Both caffeine and coffee can stimulate gastric acid secretion and decaffeinated coffee raises serum gastrin levels (17–19), even though the amount of caffeine in cola is not very high.

These findings suggest that in patients with postsurgical hypoparathyroidism, the intake of large amounts of phosphoric acid may reduce calcium absorption efficiency despite the high doses of calcium therapy, because the deficient PTH cannot balance hyperphosphatemia. However, further research is required in order to confirm the results observed.

## Concluding Remarks

The evidence of severe hypocalcemia in patients with hypoparathyroidism requires evaluation of the causal factors. In patients taking high doses of calcium without benefits, the intake of cola drinks should always be considered, and when hypoparathyroidism is also present, a low phosphorus diet is advisable.

## Author Contributions

VG, AC, GP, SB, and CG analyzed and interpreted the patient data regarding the clinical and hematological aspects. SR and MM analyzed the chemical data from Gs and Ds samples. SB and AI performed the experimental procedure introducing the probes in the stomach and duodenum. VG, SR, and SB contributed in writing the manuscript. All the authors revised and approved the final manuscript and agreed to be accountable for the content of the work.

## Conflict of Interest Statement

The authors declare that the research was conducted in the absence of any commercial or financial relationships that could be construed as a potential conflict of interest.
